# Optimization of Surface Acoustic Wave-Based Rate Sensors

**DOI:** 10.3390/s151025761

**Published:** 2015-10-12

**Authors:** Fangqian Xu, Wen Wang, Xiuting Shao, Xinlu Liu, Yong Liang

**Affiliations:** 1 Zhejiang University of Media and Communications, 998 Xueyuan Street Higher Education Zone Xia Sha, Zhejiang 310018, China; E-Mail: xufangqian2006@163.com; 2 State Key Laboratory of Acoustics, Institute of Acoustics, Chinese Academy of Sciences, No. 21, BeiSiHuan West Road, Beijing 100190, China; E-Mails: shaoxiuting10@mails.ucas.ac.cn (X.S.); liuxinlu1987@foxmail.com (X.L.); liangyong@mail.ioa.ac.cn (Y.L.)

**Keywords:** Coriolis force, delay line-oscillator, metallic dot array, partial-wave analysis, SAW rate sensor

## Abstract

The optimization of an surface acoustic wave (SAW)-based rate sensor incorporating metallic dot arrays was performed by using the approach of partial-wave analysis in layered media. The optimal sensor chip designs, including the material choice of piezoelectric crystals and metallic dots, dot thickness, and sensor operation frequency were determined theoretically. The theoretical predictions were confirmed experimentally by using the developed SAW sensor composed of differential delay line-oscillators and a metallic dot array deposited along the acoustic wave propagation path of the SAW delay lines. A significant improvement in sensor sensitivity was achieved in the case of 128° YX LiNbO_3_, and a thicker Au dot array, and low operation frequency were used to structure the sensor.

## 1. Introduction

The surface acoustic wave (SAW)-based micro rate sensor has gained increasing attraction for inertial navigation applications because it exhibits many unique properties such as superior inherent shock robustness, a wide dynamic range, low cost, small size, and long working life compared to other current gyroscope types [1]. The typical working principle of the SAW rate sensor is so-called SAW gyroscopic effect [2,3], that is, as the Coriolis force induced by the applied rotation acts on the vibrating particles along the SAW propagation path, a pseudo running wave shifted by a quarter of a wavelength will arise, and it couples with the initial SAW generated by the interdigital transducers (IDTs) on the piezoelectric substrate, resulting in the change of trajectory of the wave particles and an acoustic wave velocity shift. Consequently, a frequency signal variation proportional to the applied rotation is expected. Referring to a certain differential oscillation structure, the SAW micro rate sensor-based gyroscopic effect can be implemented. Lee *et al.* first realized a prototype of a micro rate sensor based on SAW gyroscopic effect utilizing a temperature-compensated ST quartz substrate and a differential dual-delay-line oscillator configuration [4,5], but, the corresponding sensitivity was far too low, only 0.43 Hz·deg^−^^1^·s^−^^1^. To improve the sensor sensitivity, a X-112°Y LiTaO_3_ substrate was suggested to form the SAW gyroscope because it exhibits a stronger gyroscopic effect, and a sensitivity of 1.332 Hz·deg^−^^1^·s^−^^1^ in a wide dynamic range (0~1000 deg·s^−^^1^) and good linearity are obtained [6]. Moreover, some other meaningful research works about SAW rate sensors were also reported [7,8], however, it is obvious that there is still no tangible improvement in the sensor performance because of its very weak Coriolis force.

**Figure 1:**
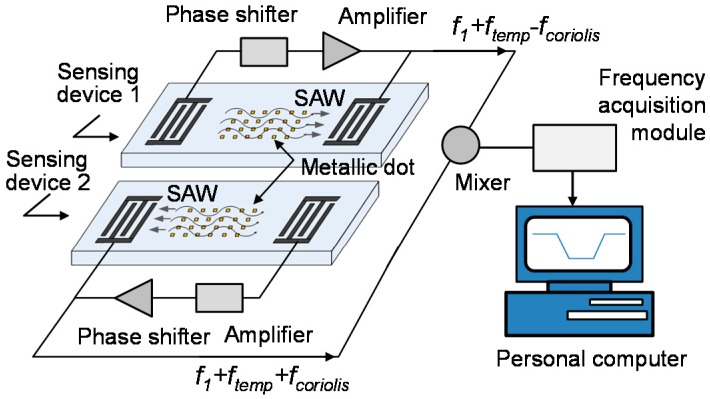
The scheme and working principle of the SAW micro rate sensor.

To achieve a breakthrough in the performance of the SAW-based rate sensor, a creative idea was proposed whereby a metallic dot array strategically deposited on the SAW propagation path of SAW devices was considered to enhance the Coriolos force acting on the propagating SAW [9,10,11]. The scheme of the proposed SAW rate sensor incorporating a metallic dot array is depicted in Figure 1. The proposed sensor was composed of differential delay line-oscillators set in opposite directions, and metallic dot arrays deposited along the SAW propagation path of each SAW device. The centre distance of the dot element in the array is set to one wavelength in each direction, and also, the size of dots is a quarter-wave. When the sensor is subjected to an angular rotation, the Coriolis force acts on the vibrating metallic dots because of the Coriolis effect (*F*_coriolis_ = 2*m*(*v* × Ω); *m*: mass of dot, *v*: velocity of the dot, Ω: rotation rate). Moreover, the direction of the force is the same as the direction of wave propagation. Therefore, the amplitude and velocity of the wave are changed (Δ*v*_c_), and this change induces a shift in the oscillation frequency (Δ*f*_c_). Obviously, the enhanced Coriolis force will improve significantly the detection sensitivity. Exciting detection sensitivity results (16.7 Hz·deg^−^^1^·s^−^^1^) were achieved with a 80 MHz rate sensor on X-112°Y LiTaO_3_ with a 900 nm Cu dot array distribution [9]. This provides a good start to break through the detection sensitivity bottleneck of SAW-based rate sensors, even though there is still a great gap between the obtained sensitivity and the demands for real applications.

The main purpose of this work is to determine the optimal design parameters by analyzing the partial-wave in layered media utilizing the acoustic wave equation considering the contribution of the Coriolis force [12]. The materials for the piezoelectric crystal and metallic dots, dot geometry, and operation frequency were determined theoretically. The theoretical predictions were readily confirmed in rate sensing experiments by using the sensor scheme mentioned in Figure 1. Higher sensitivity and good linearity were achieved by using the 128°YX LiNbO_3_, a thick Au dot array and lower operation frequency.

## 2. Theoretical Determination of Design Parameters

In this section, to simplify the theoretical analysis process, the metallic dot is considered as a semi-infinite surface, hence, the pre-rotated SAW propagation along the piezoelectric substrate with metallic dot distribution was analyzed by solving the partial-wave equations in layered structure described in our previous work [11], and the SAW propagates along the *x*(*x*_1_) axis on the *x*-*y*(*x*_2_)-plane at *z*(*x*_3_) = 0. The key factors which influence the rate sensor performance were studied theoretically. Hence, the optimal design parameters were extracted.

### 2.1. Theoretical Model

**Figure 2:**
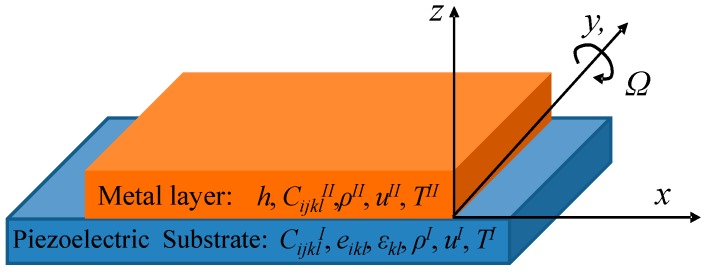
The coordinate system used in this study.

Considering there is an anisotropic and piezoelectric medium occupying a half-space (*x*_3_ ≤ 0) with interdigital transducer (IDT) about the plane (*x*_3_ = 0) and a metallic layer (0 ≤ *x*_3_ ≤ *h*), as schematically illustrated in Figure 2, the dynamic wave equations considering the Coriolis force contribution of linear piezoelectricity in half-space piezoelectric substrate take the following forms in this coordinate system mentioned in Figure 2:

(1) $$\left\{ \begin{array}{l} {C_{ijkl}}^{I}u_{k,jl}+e_{kij}\varphi_{,jk}=\rho^{I}[{u_{i}}^{I}+2\varepsilon_{ijk}\Omega_{j}{u_{k}}^{I}-\left(\Omega_{j}^{2}{u_{i}}^{I}-\Omega_{i}\Omega_{j}{u_{j}}^{I}\right)]\\ e_{jkl}{u_{k,jl}}^{I}-\varepsilon_{jk}\varphi_{,jk}=0 \end{array}\right.$$

where Einstein’s summation rule is used, and the indices changed from 1 to 3. We denote by *u_i_^I^* the mechanical displacements and by φ the electric potential. *c_ijkl_^I^*, *e_kij_*, and *ε_ij_^I^* stand for the elastic, piezoelectric and dielectric constants, and *ρ^I^* for the mass density of the piezoelectric substrate, respectively. *ε_ijk_* is the Levi-civita symbol. We assume a general solution of Equation (1), the particle displacement and electrical potential, are in the form:

(2) $$\left\{ \begin{array}{l} {u_{i}}^{I}={A_{i}}^{I}\exp[-j(\omega t-\beta x_{1}-\beta \alpha x_{3}\text{)]}\\ \varphi ={A_{4}}^{I}\exp[-j(\omega t-\beta x_{1}-\beta \alpha x_{3}\text{)]} \end{array}\right.$$

where *β* and ω are the wave numbers in the *x*_1_ direction and the angular frequency, respectively. α is a decay constant along the *x*_3_ direction. *A_i_^I^* (*i* = 1, 2, 3) and *A*_4_*^I^* are wave amplitudes. Substitution of Equation (2) into Equation (1) leads to four linear algebraic equations (Cristoffel equation) for *A_i_^I^* and *A*_4_*^I^*, that is:

(3) $$\left[\begin{array}{cccc} \Gamma_{11} & \Gamma_{12} & \Gamma_{13} & \Gamma_{14}\\ \Gamma_{21} & \Gamma_{22} & \Gamma_{23} & \Gamma_{24}\\ \Gamma_{31} & \Gamma_{32} & \Gamma_{33} & \Gamma_{34}\\ \Gamma_{41} & \Gamma_{42} & \Gamma_{43} & \Gamma_{44} \end{array}\right]\left[\begin{array}{c} A_{1}\\ A_{2}\\ A_{3}\\ A_{4} \end{array}\right]=0$$

Then, for nontrivial solutions of *A_i_^I^* and/or *A*_4_*^I^*, the determinant of the coefficient matrix of the linear algebraic equations must vanish, and this leads to a polynomial equation of degree eight for α. The coefficients of this polynomial equation are generally complex. To ensure the decrease in the displacement *u_i_* and the potential φ into the substrate, the generally complex constant α must have a negative imaginary part. Thus, we select four eigenvectors with negative imaginary part denoted by α*_n_* (*n* = 1, 2, 3, 4), and the corresponding eigenvectors by *A_i_^I^*^(*n*)^ *=* [*A*_1_*^I^*^(*n*)^ *A*_2_*^I^*^(*n*)^ *A*_3_*^I^*^(*n*)^ *A*_4_*^I^*^(*n*)^], *n* = 1, 2, 3, 4. Thus, the general wave solution to Equation (1) in the form of Equation (2) can be written as:

(4) $$\{\begin{array}{c} u_{i}=\sum_{n=1}^{4}A_{i}^{\left(n\right)}{C_{n}}^{I}\exp\{-jk_{s}(x_{1}+a_{n}x_{3})\}\\ \varphi =\sum_{n=1}^{4}A_{4}^{\left(n\right)}{C_{n}}^{I}\exp\{-jk_{s}(x_{1}+a_{n}x_{3})\} \end{array},i=1,2,3$$

where *C_n_^I^* (*n =* 1, 2, 3, 4) are the weight factors, and can be determined by the boundary condition.

Then, the acoustic wave equation considering the contribution of Coriolis force in the isotropic metal layers is:

(5) $${C_{ijkl}}^{II}u_{k,jl}=\rho^{II}[{{\ddot{u}}_{i}}^{II}+2\varepsilon_{ijk}\Omega_{j}{{\dot{u}}_{k}}^{II}-(\Omega_{j}^{2}{u_{i}}^{II}-\Omega_{i}\Omega_{j}{u_{j}}^{II})\text{], }i,j,k,l\text{=1,2,3}$$

Here, ρ*^II^* is the density of the metal layer, *u_i_^II^* is the component of the acoustic wave displacement. The solution of Equation (3) is assumed as:

(6) $${u_{i}}^{II}={A_{i}}^{II}\exp[-j(\omega t-\beta x_{1}-\beta \eta x_{3}\text{)]}$$

Substituting the Equation (4) into Equation (2), the corresponding Cristoffel equation in metal layer can be written as:

(7) $$\left[\begin{array}{ccc} \Gamma_{11} & \Gamma_{12} & \Gamma_{13}\\ \Gamma_{21} & \Gamma_{22} & \Gamma_{23}\\ \Gamma_{31} & \Gamma_{32} & \Gamma_{33} \end{array}\right]\left[\begin{array}{c} A_{1}\\ A_{2}\\ A_{3} \end{array}\right]=0$$

Also, for nontrivial solutions of *A_i_^II^*, the determinant of the coefficient matrix of the linear algebraic equations must vanish, and there is an algebraic equation of the 6-th order in η*.* Then, substituting the four eigenvectors into Equation (3), the corresponding normalized amplitude *A_i_^II(n)^* can be determined. Full solution of the wave equation was the linear combination of four basic groups:

(8) $$u_{i}^{I}=\sum_{n=1}^{6}C_{n}^{\text{I(n)}}\exp\left[-j\left(\omega t-\beta x_{1}-\beta \eta_{n}^{I}x_{3}\right)\right]$$

where the coefficient $C_{n}^{Ι}$ was determined by the boundary conditions.

Then, the solutions of the motion equations should satisfy both the mechanical boundary condition and the electrical boundary condition respectively. The mechanical boundary condition and electrical boundary condition at boundary between the piezoelectric substrate and metal layer (*x*_3_ *=* 0) and boundary between metal layer and vacuum (*x*_3_ = *h*) as schematically illustrated in Figure 2 are:

(9) $$\begin{array}{l} \left\{\begin{array}{c} T_{i3}^{I}-T_{i3}^{II}=0\\ u_{i}^{I}-u_{i}^{II}=0\\ \varphi^{II}=0 \end{array},i=1,2,3\right\}x_{3}=0\\ \left\{T_{i3}^{II}=0,\;i=1,2,3\right\}x_{3}=h \end{array}$$

Then, substituting the solutions of the wave Equations (4) and (8) into the boundary conditions Equation (9), the following equation can be obtained:

(10) $$H_{m}C_{m}=0$$

The condition of nontrivial solution in Equation (10) was the determinant coefficient should be zero, that is:

(11) $$\left|H_{m}\right|=0$$

To simplify the theoretical calculation, the iteration method was used referring to the Matlab software. Based on the deduced formulas, the SAW velocity shift depending on the normalized rotation can be computed.

### 2.2. Numerical Results and Discussion

**Figure 3:**
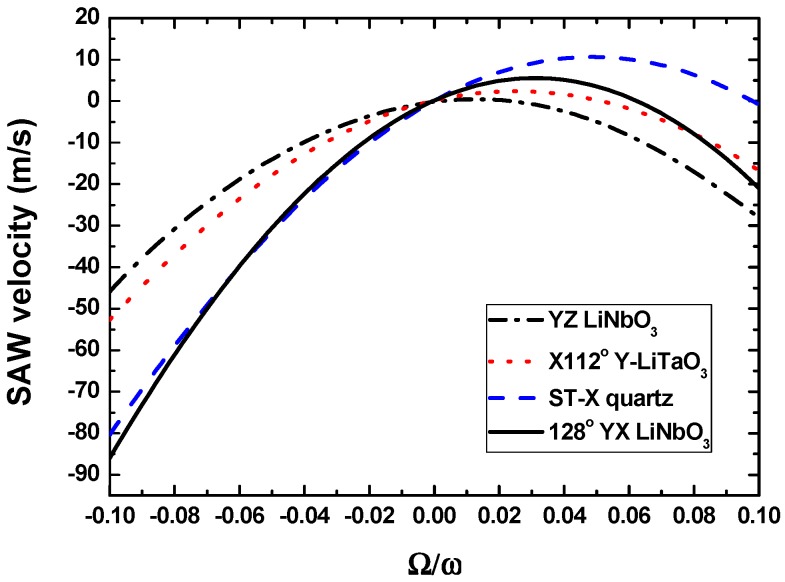
Gyroscopic effect in various piezoelectric substrates without metallic dots.

The piezoelectric crystals used for the SAW sensors analyzed herein are YZ-LiNbO_3_, X-112°Y LiTaO_3_, ST-X quartz, and 128°YX LiNbO_3_, and the metallic dot materials are assumed as copper (Cu) and gold (Au), respectively. The corresponding mechanical parameters are listed in Table 1. In the calculation, the piezoelectric crystals are assumed to be rotated around *y*-axis, and the SAW along the substrate propagates along the *x*-axis (Figure 2). Figure 3 illustrates the gyroscopic effect in above piezoelectric crystals without metallic dot array distribution. ST-X quartz and 128°YX LiNbO_3_ exhibit larger gyroscopic effects than other materials in the relative rotation range of −0.02~0.02.

**Table 1:** Mechanical parameters for the piezoelectric substrate and metallic dots [13,14].

Materials	Euler Angle	Stiffness Coefficients (10^10^ N/m^2^)	Piezoelectric Modules (C/m^2^)	Permittivity Constants (10^−12^ F/m)	Density (kg/m^3^)
YZ LiNbO_3_	(0°, 90°, 90°)	C_11_: 23.3 C_33_: 27.5 C_44_: 9.4 C_12_: 4.7 C_13_: 8.0 C_14_: −1.1	e_15_: 2.58 e_22_: 1.59 e_31_: −0.24 e_33_: 1.44	ε_11_: 51 × ε_0_ ε_33_: 43 × ε_0_ ε_0_: 8.854	7450
128°YX LiNbO_3_	(0°, 37.86°, 0°)				
ST-X quartz	(0°, 132.75°, 0°)	C_11_:8.674 C_12_: 0.699 C_13_:1.191 C_14_: −1.791 C_33_:10.72 C_44_: 5.794	e_x1_: 30.171 e_x4_: −0.0436 e_z6_: 0.14	ε_11_: 4.5 × ε_0_ ε_33_: 4.6 × ε_0_ ε_0_: 8.854	2651
X-112°Y LiTaO_3_	(90°, 90°, 112.2°)	C_11_: 23.28 C_12_: 4.65 C_13_: 8.36 C_14_: −1.05 C_33_: 27.59 C_44_: 9.49	e_x5_: 2.64 e_y2_: 1.86 e_z1_: −0.22 e_z3_: 1.71	ε_11_:40.9 × ε_0_ ε_33_:42.5 × ε_0_ ε_0_: 8.854	7454
Cu		C_11_: 17.69 C_33_: 7.96			8900
Au		C_11_:18.6 C_12_: 15.7 C_44_: 4.2			19,300

**Figure 4:**
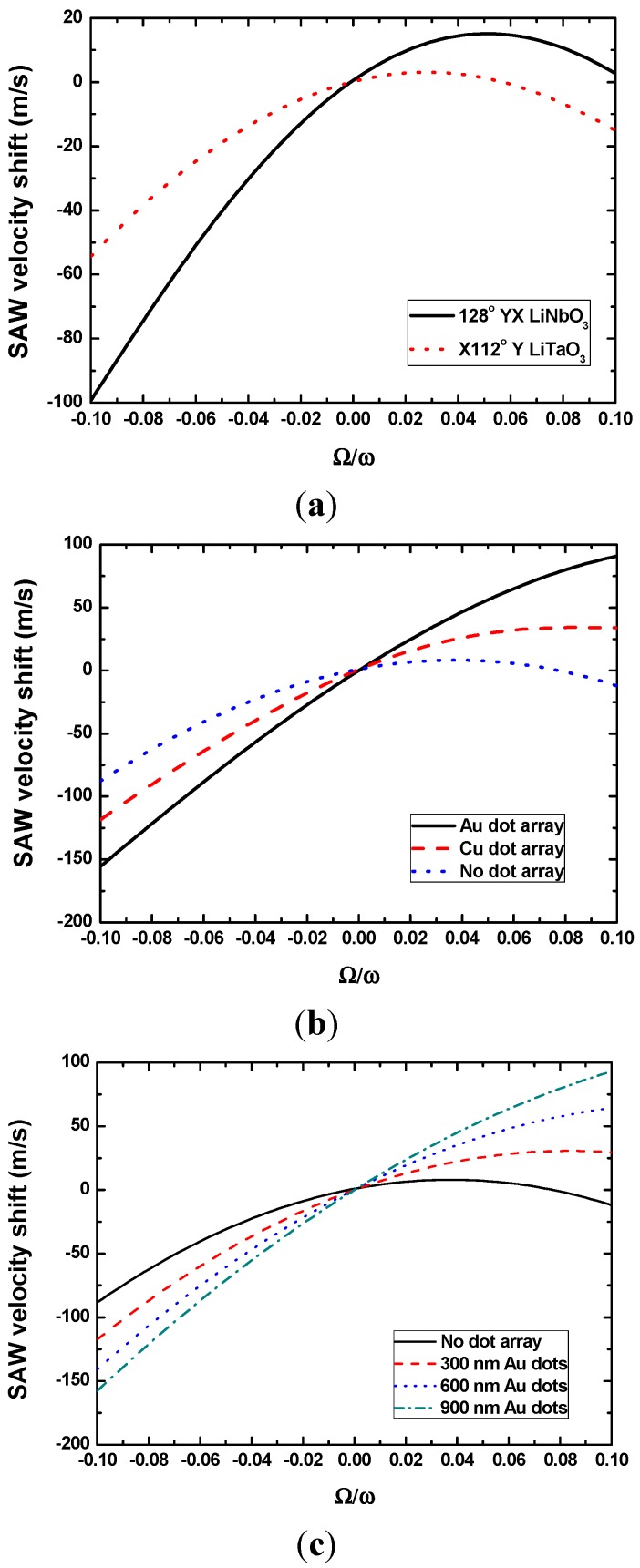
Calculated effects from the piezoelectric substrate (**a**), metallic dots (**b**), and geometry of dots (**c**).

Additionally, to compare the gyroscopic effect in various piezoelectric substrates with metallic dot array distributions, the SAW velocity shift induced by the external rotation was calculated in the case of X-112°Y LiTaO_3_ and 128°YX LiNbO_3_ with 900 nm thick Cu dots, as shown in Figure 4a. It is obvious that the 128°YX LiNbO_3_ shows a stronger gyroscopic effect compared to X-112°Y LiTaO_3_. Also, the contributions from the various dot materials (900 nm Au dot and Cu dot) are studied in Figure 4b in case where 128°YX LiNbO_3_ was applied, and it is clear that a heavy metallic dot induces a larger SAW gyroscopic effect; hence, high sensor sensitivity will be expected. Similarly, increasing the metallic dot thickness can also improve the gyroscopic effect, as depicted in Figure 4c, in the case where various Au thicknesses from 300 nm to 900 nm are applied on 128°YX LiNbO_3_. Moreover, it is easy to make a conclusion in the above calculation that decreases in sensor operation frequency at a given rate angular will increase the SAW velocity shift, that is, the sensor sensitivity will be improved by decreasing the sensor operation frequency.

## 3. Sensor Experiments

### 3.1. Physical Structure of the SAW Rate Sensor

**Figure 5:**
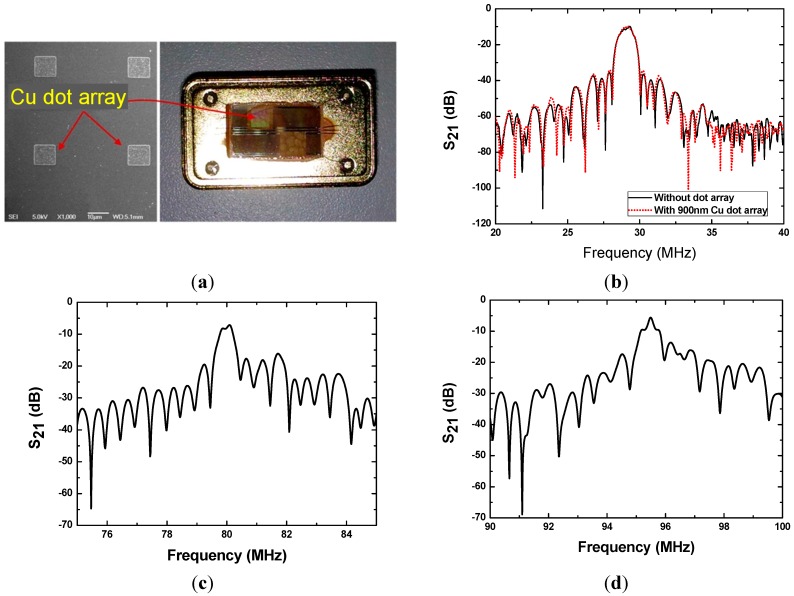
Developed SAW sensor chip (**a**), measured S_21_ of 30 MHz SAW device (**b**), and 80 MHz SAW device (**c**), and 95 MHz SAW device (**d**).

A series of SAW rate sensors utilizing various design parameters listed in Table 2 were constructed to confirm the theoretical predictions. The sensor scheme is depicted in Figure 1, in which, two parallel SAW delay lines with opposite directions and metallic (Cu and Au) dot array distribution were fabricated on a same piezoelectric substrate by a photolithography technique. Single phase unidirectional transducers (SPUDTs) and combed transducers were the structures used in the SAW delay lines to reduce the insertion loss and improve the frequency stability of the oscillator [15]. Using a HP 8753D network analyzer, the amplitude responses (S_21_) of the developed SAW delay lines (Figure 5a) were measured under matched conditions. Figure 5 shows the typical S_21_ plots from the SAW devices on 128°YX LiNbO_3_ with 900 nm thick Au dots and operation frequency of 30 MHz, 80 MHz, and 95 MHz, respectively. It is worth noting that the effect of the metallic dot array distribution on device performance is insignificant and can be neglected (Figure 5b).

**Table 2:** Design parameters for the SAW sensor chips.

Items	Design Parameters
Piezoelectric substrates	X-112°Y LiTaO_3_, 128°YX LiNbO_3_
Operation frequency	95 MHz, 80 MHz, 30 MHz
Metallic dot materials	Cu, Au
Metallic dot thickness	300 nm, 600 nm, 900 nm
Metallic dot size	1/4λ × 1/4λ

**Figure 6:**
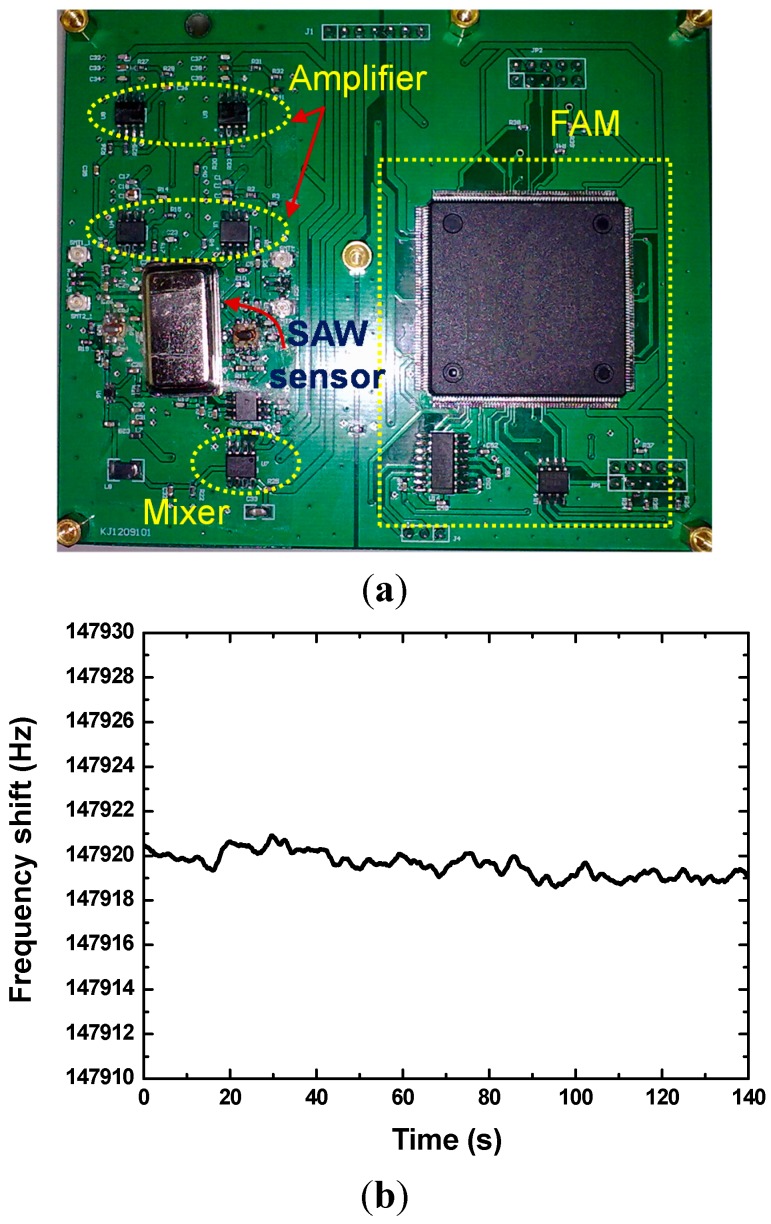
The PCB for the developed SAW sensor (**a**), and short-term frequency stability testing of SAW oscillator (**b**).

Next, the fabricated SAW chips were loaded in a standard metal base (Figure 5a), and acted as the oscillation feedback elements. The launching and readout transducers of the SAW devices were connected by an oscillation circuit composed of a discrete elements (amplifier, phase shifter, mixer and LPF) on a printed circuit board (PCB). The outputs of the oscillators were mixed to obtain a differential frequency in kHz range. This technique allows doubling the detection sensitivity and a reduction of the influence of the thermal expansion of the substrate. The differential frequency signals was picked up by the frequency acquisition module (FAM) on the PCB and output to a PC, as shown in Figure 6a. To further improve the frequency stability of the oscillator, the oscillation was modulated at the frequency point with lowest insertion loss by a strategically phase modulation [16]. The typical short term frequency stability of the oscillator at room temperature (20 °C) is characterized as 0.8 Hz/s, as shown in Figure 6b.

### 3.2. Sensor Experiments and Discussions

**Figure 7:**
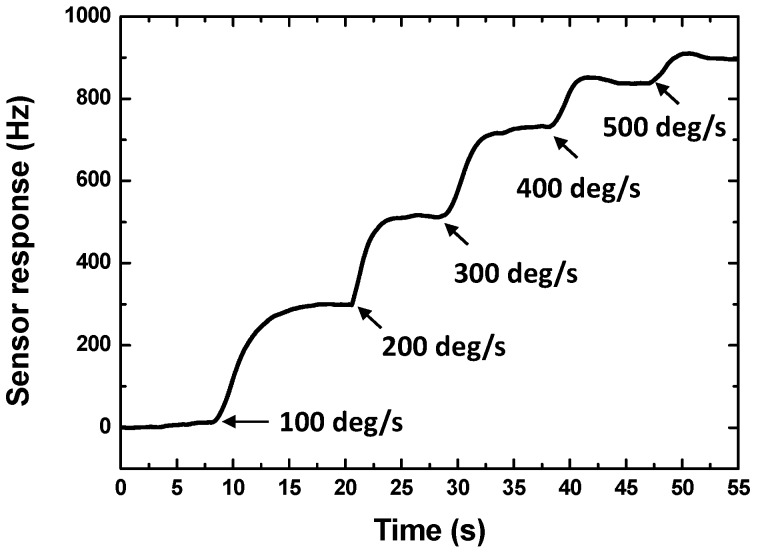
The continuous response of the stimulate 95 MHz sensor on X-112°Y LiTaO_3_ with 300 nm thick Cu dot array.

**Figure 8:**
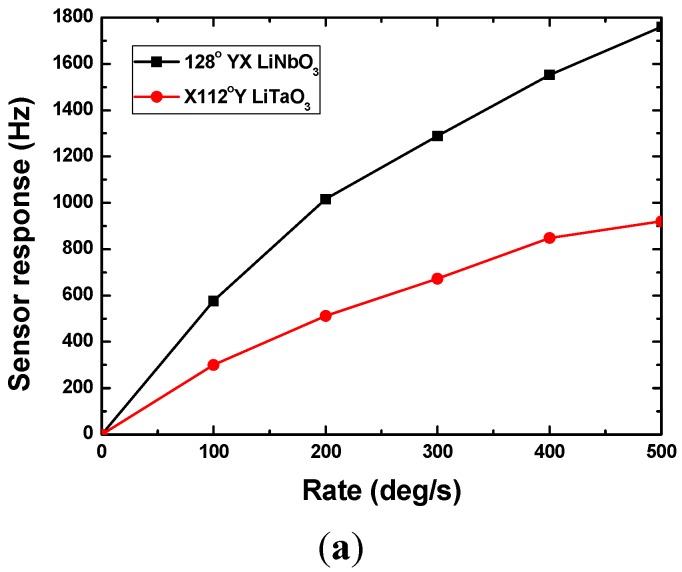

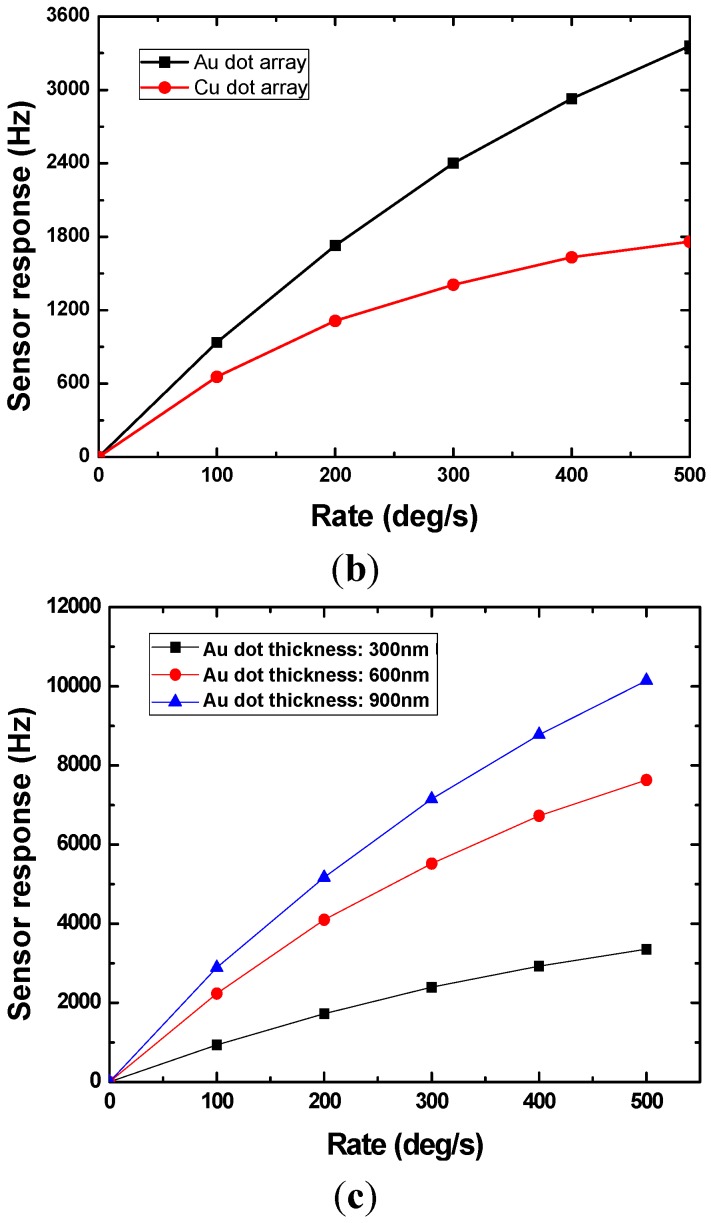
Gyroscopic effect comparison among various piezoelectric substrate (**a**), metallic dot material (**b**), and dot thickness (**c**), sensor operation frequency: 95 MHz.

**Figure 9:**
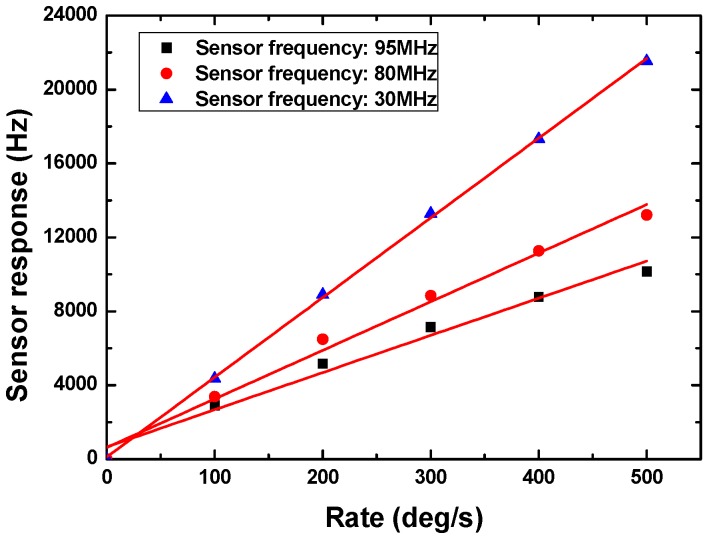
The experimental sensor response depending on various operation frequencies, piezoelectric substrate: 128°YX LiNbO_3_, Au dot thickness: 900 nm.

Next, the sensor performance of the packaged SAW micro rate sensors were evaluated experimentally by using a precision temperature-controlled rate table. A self-made interface display program was used to record and plot the sensor responses in real time. The rate sensor is subjected to a rotation in the *y*-axis. Figure 7 shows the continuous response of the stimulate sensor on X-112°Y LiTaO_3_ with 300 nm thick Cu dot array. The data sampling time of the FAM is 18 ms, which means the FAM collects experimental data every 18 ms, so that one point in Figure 7 corresponds to a 18 ms interval. Next, the response of 95 MHz rate sensors on 128°YX LiNbO_3_ and X-112°Y LiTaO_3_ in case of 300 nm Cu dot array were tested as shown in Figure 8a. It is clear that the 128°YX LiNbO_3_ displays a larger gyroscopic effect, which agrees well with the theoretical calculation in Figure 5a. Thus, the 128°YX LiNbO_3_ substrate is adopted in the following experiments. Figure 8b illustrates the effect from the metallic dot materials on sensor response; obviously, the Au dot will provide the strongest sensor response. Also, thicker dots will obtain larger sensor responses, as shown in Figure 8c, where Au dots with thicknesses of 300 nm, 600 nm, and 900 nm are used. Moreover, the effect from the operation frequency on sensor response is also analyzed experimentally, as described in Figure 9. With the decrease of the sensor operation frequency, the sensor sensitivity increases, and the highest sensitivity was observed from the rate sensor on 128°YX LiNbO_3_ substrate with 900 nm Au dots and operation frequency of 30 MHz. All the measured results indicate the validity of the theoretical predictions. The measured detection frequency is evaluated as ~43 Hz·deg^−1^·s^−1^, and good linearity was observed in the dynamic range of 0~500°/s. The measured sensitivity is over 2.7 times larger than that of reported similar rate sensors [9].

**Figure 10:**
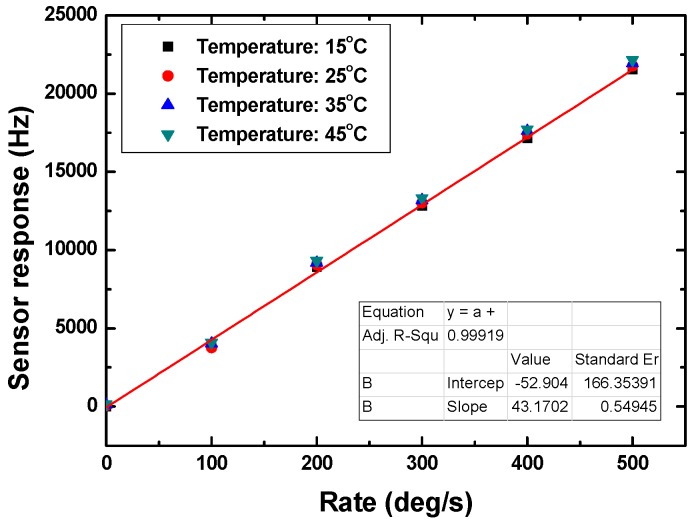
Testing of temperature effect on sensor response.

Additionally, due to the differential oscillation structure, the temperature effect is compensated well as shown in Figure 10. The changes in the detection sensitivity at various temperatures were less than 5% (the detection sensitivities at temperature of 15 °C, 25 °C, 35 °C and 45 °C are 43.17 Hz·deg^−^^1^·s^−^^1^, 43.83 Hz·deg^−^^1^·s^−^^1^, 43.96 Hz·deg^−^^1^·s^−^^1^, and 44.26 Hz·deg^−^^1^·s^−^^1^, respectively). This implies that the temperature effect was effectively removed by using the differential oscillation structure.

## 4. Conclusions

An optimization of a SAW rate sensor was performed by using the method of partial-wave analyses in layered media. The optimal design parameters were determined theoretically. The theoretical predictions were confirmed well in the subsequent rate sensing experiments. Significant improvements of the sensor performance were observed with the optimized SAW rate sensor. Higher sensitivity of ~43 Hz·deg^−^^1^·s^−^^1^ and good linearity in larger dynamic range of 0~500 Hz were achieved with the developed SAW rate sensor adopting 128°YX LiNbO_3_, 900 nm thick Au dots and an operation frequency of 30 MHz.
